# Case Report: A Clinical and Genetic Analysis of Childhood Growth Hormone Deficiency With Familial Hypercholesterolemia

**DOI:** 10.3389/fendo.2021.691490

**Published:** 2021-06-18

**Authors:** Shengmin Yang, Xiaoan Ke, Hanting Liang, Ran Li, Huijuan Zhu

**Affiliations:** Key Laboratory of Endocrinology of National Health Commission, Department of Endocrinology, State Key Laboratory of Complex Severe and Rare Diseases, Peking Union Medical College Hospital, Chinese Academy of Medical Science and Peking Union Medical College, Beijing, China

**Keywords:** growth hormone deficiency, familial hypercholesterolemia, LDL-C, GH, IGF-1

## Abstract

**Background:**

Growth hormone deficiency (GHD) is a developmental disorder in children characterized by low growth hormone (GH), short stature and unfavorable lipid profiles. Familial hypercholesteremia (FH) is an inborn disorder of low-density lipoprotein cholesterol (LDL-C) metabolism which results in premature cardiovascular events. The co-occurrence of GHD and FH, which may aggravate the hypercholesteremic condition in the affected individuals, had rarely been discussed in previous publication.

**Methods:**

This work reports two cases of GHD with FH, and explores the lipid profiles of GHD children and their therapeutic response to recombinant human growth hormone (rhGH). The diagnosis of GHD is based on low peak GH level (<7 ng/mL) in GH provocation test. FH is diagnosed by high LDL-C level (≥ 4 mmol/L) and confirmed genetic mutations in the LDL-C metabolic pathway. We also searched all previously published metabolic studies on GHD children as of December 31, 2020. Information on their LDL-C, duration and dose of rhGH treatment were retrieved and summarized.

**Results:**

The first case was a 5.3 year-old boy. His height was 103.6 cm (SDS = -2.29) and his peak GH in provocative test was 6.37 ng/mL. Additionally, his LDL-C was 4.80 mmol/L and he harbored a heterozygous mutation for the *apolipoprotein B (APOB)* gene (c.10579 C > T). The second case was a 9-year-old girl at the height of 117.3 cm (SDS = -2.91). Her GH peaked at 4.99 ng/mL in insulin-induced hypoglycemic test and 2.80 ng/mL in L-dopa test. Her LDL-C was 6.16 mmol/L, and she carried a mutated copy of the *low-density lipoprotein receptor (LDLR)* gene (c.809 G > A). Literature review indicated that GHD children suffered from higher baseline LDL-C, but it was significantly reduced after rhGH treatment.

**Conclusions:**

FH should be considered if a GHD child has remarkably elevated LDL-C that cannot be attributed to low GH level alone. Genetic mutations in the LDL-C metabolic pathway prevent the body from effectively metabolizing lipids, thereby resulting in early-onset hypercholesteremia and probably playing a negative role in children’s growth.

## Introduction

Childhood-onset Growth hormone deficiency (GHD) is closely related to dyslipidemia, increased carotid intima-media thickness, abdominal obesity and impaired left ventricular diastolic function (1). Moreover, the lipid profiles of GHD children can be worsen if they also suffer from familial hypercholesteremia (FH). FH is an early onset genetic disease characterized by persistently elevated low-density lipoprotein cholesterol (LDL-C), which predisposes patients to premature cardiovascular diseases (CVDs). FH is commonly caused by mutations in one of the four genes in the LDL-C metabolic pathway, namely *low-density lipoprotein receptor (LDLR)*, *apolipoprotein B (APOB)*, *proprotein convertase subtilisin/kexin type 9 (PCSK9)* and *low density lipoprotein receptor adaptor protein 1 (LDLRAP1)* (2, 3). FH affects 1/500 people worldwide, while China accounts for 8% of FH diagnosed (4). Early diagnosis followed by lifelong cholesterol-lowering therapy is crucial for prolonging CVD-free survival. FH children typically have untreated LDL-C ≥ 4 mmol/L, plus positive family history of early-onset CVDs or elevated LDL-C (5). The diagnosis of FH can be further confirmed by genetic tests that highlight the causative mutations (6). Statins, which are recommended as first-line treatment for FH, significantly curb the progression of subclinical atherosclerosis and reduce the risk of CVDs in FH patients (7). Recombinant human growth hormone (rhGH) therapy reduces LDL-C and total cholesterol (TC) in both GHD children and heterozygous FH (HeFH) patients in a dose and duration dependent manner by enhancing LDL receptor activity in the liver (8–10). Therefore, the administration of rhGH to GHD children with FH may reduce the dose of statins needed to control blood cholesterol. Here, we report two cases of HeFH patients who first visited the hospital for GHD.

## Materials and Methods

### Participants

In this study, 2 probands with coexisting GHD and FH were included. The patients underwent routine examinations to differentiate the cause of growth deficiency and hypercholesteremia, including blood routine, urine routine, thyroid function, gonadal function, GH, serum insulin-like growth factor-1 (IGF-1) and GH stimulation tests. Blood samples were collected in the morning after an overnight fast, and a solid-phase two-site chemiluminescent immunometric assay (IMMULITE 2000, Siemens, UK) was performed to quantify IGF-1 and baseline GH. The results for GH were calibrated against the international rhGH standard 98/574 (11). With regard to the GH stimulation tests, the first case was assessed by the combined simultaneous arginine-clonidine test, while the second case took the insulin-induced hypoglycemia GH provocative test and the L-dopa GH provocative test. The arginine-clonidine test was performed by intravenously infusing arginine at a dose of 0.5 g/kg (maximum dose allowed: 30g in total) over 30 minutes and orally administering clonidine at a dose of 0.15mg/m^2^ after an overnight fast. Blood samples were obtained before the arginine infusion and every 30 minutes for up to 180 minutes after the infusion. In terms of the insulin-induced hypoglycemia test, blood was consecutively drawn at 30, 60, 90 and 120 minutes after the subcutaneous injection of insulin at a dose of 0.1 U/kg. As for the L-dopa GH provocative test, the dose of L-dopa was dependent on the patient’s weight. Specifically, the dose of L-dopa was 125 mg for patients below 10kg, 250 mg for patients weighing 10-30 kg, and 500 mg for those weighing more than 30 kg. Blood was sampled before medication, and at 30, 60, 90, and 120 minutes following L-dopa administration. For the IMMULITE 2000 (Siemens) assay, Wagner et al. recommended a peak GH cutoff at 7.77 ng/mL for diagnosing pediatric GHD (12). We adopted a more rigorous GH threshold of 7 ng/mL in this study. The Pediatric Endocrine Society recommended estrogen-priming for boys above 11-year-old and girls above 10-year-old (13). However, both of our patients were too young to receive the primed test. The patients also underwent plain X ray of the left hand and wrist to assess the bone age. Informed consent was obtained from all patients and their parents.

Whole exome sequencing (WES) was performed according to the manufacturer’s standard procedure (MagPure Buffy Coat DNA Midi KF Kit, D3537-02, MAGEN) to identify mutated genes which may be responsible for high blood cholesterol and/or growth deficiency. Firstly, genomic DNA was extracted from 2ml of peripheral blood drawn from each proband and his/her parents. Next, the genomic DNA was broken into 100- to 500-bp fragments *via* BGI’s enzyme kit (Segmentase; BGI), among which fragments at the length of 280- to 320-bp were captured by magnetic beads. Then, a DNA library was constructed through ligation-mediated polymerase chain reaction. Afterwards, the library was incubated for 16 to 24 hours at 47°C to allow for adequate array hybridization (Roche NimbleGen). The Agilent 2100 Bioanalyzer system and BMG microplate reader were used to estimate the magnitude of the products’ enrichment. Finally, sequencing was performed by the PE100+100 set in MGISEQ-2000. After mapping clear reads filtered by the existing criteria (14) to the human genome reference (hg19) with the BWA (Burrows-Wheeler Aligner) Multi-Vision software package (15), we used the GATK software (16) to detect single nucleotide variants (SNVs), insertions, and deletions. The mutations identified by WES were validated by Sanger sequencing of DNA extracted from both patients and their parents.

### Literature Review

We searched for previously published papers on the lipo-metabolic profiles of GHD children treated with rhGH from PubMed, Embase and Cochrane Central Register of Controlled Trials from January 1, 1990 to December 31, 2020 (detailed search strategy shown in **Supplementary Table 1**). Statistical analysis of the lipid profiles of GHD children was performed by the R package ‘metafor’ (17). As lipid data were continuous, the differences were estimated using standard mean difference (SMD) with estimated 95% confidence interval (CI). Heterogeneity was assessed by the chi-square test and the I^2^ statistic. The random-effects model was used regardless of the I^2^ value. A two-sided P < 0.05 was considered to be statistically significant. The studies were selected based on the following criteria: 1) participants: children aged < 18 years old during the study; 2) disease: GHD; 3) intervention: rhGH replacement therapy; 4) study design: controlled clinical trial, irrespective of randomization, blindness and the type of control group; and 5) language of publication: English. Trials in which the LDL-C of subjects in the control group was not measured at the end of the study were excluded from the analysis.

Two investigators (SY and XK) independently extracted all data using standard data collection forms. Data were recorded for the participant characteristics (age, sample size), intervention (dose and duration of rhGH treatment) and outcome data (LDL-C level at baseline and the end of the study). Any disparities were resolved by consensus and scrutinized by a senior researcher (Prof. Huijuan Zhu).

## Results

### Case Presentation

**Figure 1:**
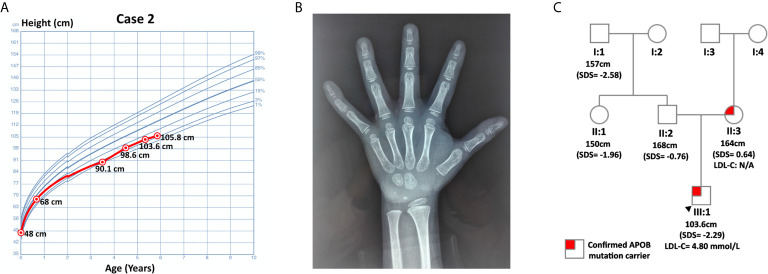
Information on case 1. **(A)** Growth curve. **(B)** Plain X-ray of left hand and wrist. **(C)** Pedigree.

Case 1 was a 5-year and 4-month-old boy who was presented at the hospital for short stature. He was born at full term with normal birth measurements: length 48 cm, weight 3.08 kg. He was normal in teething, walking, speaking and intelligence as compared with age and sex matched children. Physical examination revealed proportionate short stature, normal facial features and limbs, and normally developed prepubertal external genitalia (Tanner stage I). He was 103.6 cm (SDS= -2.29) in height and 15 kg (SDS= -2.07) in weight. His BMI was 13.98 kg/m^2^ (SDS = -0.98). His bone age was assessed to be 2-3 years old, which was about 3 years behind the chronological age. His thyroid function, fasting blood glucose (4.36 mmol/L) and 25-hydroxyvitamin D [25(OH)D] level were normal. Magnetic resonance imaging (MRI) of the pituitary showed no abnormalities. The peak GH in arginine-clonidine GH provocative test (6.37 ng/mL) was lower than 7 ng/mL, indicating the diagnosis of GHD (12). The diagnosis of GHD was further validated by his IGF-1 level and growth pattern. In particular, his IGF-1 (56.5 ng/mL, SDS = -1.89 ~ -1.85, normal range: 50-286 ng/mL for 5-year-old children, 52-297 ng/mL for 6-year-old children) and IGF binding protein-3 (IGFBP3, 3.29 ug/mL, age-specific normal range: 2.65-7.89 ug/mL, SDS = -1.48) were notably lower relative to his peers. Buoquete et al. demonstrated that a considerable proportion of GHD children had normal IGF-1 and IGFBP-3. Through receiver-operator curve (ROC) analysis, the best IGF-1 SDS threshold for distinguishing GHD and idiopathic short stature (ISS) was -1.65 (sensitivity: 68%, specificity: 97%) (18). As the patient’s IGF-1 was below -1.85 SDS, he was more likely to suffer from GHD rather than ISS. With regard to his growth curve, further follow-up revealed that he grew to 105.8 cm (SDS = -2.39) in height and 17 kg (SDS = -1.58) in weight at 5-year-10-month-old (**Figure 1**). Namely, he only gained 2.2 cm in 6 months, which implied progressive growth retardation. The diagnosis of GHD in this patient is substantiated by the following evidence: low GH peak in arginine-clonidine test, IGF-1 SDS < -1.65, a delayed bone age at diagnosis, as well as progressive growth restriction (19).

Routine laboratory tests showed elevated TC (7.08 mmol/L, hypercholesterolemia: ≥ 5.18 mmol/L, ideal: < 4.4 mmol/L) and LDL-C (4.80 mmol/L, hypercholesterolemia: ≥ 4 mmol/L, ideal: < 2.85 mmol/L) (6). His triglyceride (TG, 0.46 mmol/L, abnormal: ≥ 1.70 mmol/L) and high-density lipoprotein cholesterol (HDL-C, 2.11 mmol/L, abnormal: ≤ 1.04 mmol/L) were normal. WES did not detect any genetic mutations that could account for GHD in this patient. Nevertheless, it identified a pathogenic variant of *APOB*: c.10579 C > T (p. Arg3527Trp, Ensembl transcript ID of the reference sequence: NM_000384.2), which was commonly observed in Asian FH patients (20). This finding was further confirmed by Sanger sequencing using DNA samples from this patient and his parents. The patient inherited this missense mutation from his mother, while his father did not harbor any mutations at this site (**Figure 1**).

Case 2 was a 9-year-old girl who visited the endocrinology unit for growth retardation. Her body length (50 cm) and weight (3.6 kg) were normal at birth. Plain X-ray film indicated a bone age of 6-year, which was 3 years behind the actual age. Karyotype testing showed normal female karyotype (46XX). At age 9, her height was 117.3 cm (SDS = -2.91). Her weight was 22.5 kg (SDS = -1.41), and her BMI was 16.35 kg/m^2^ (SDS = 0.44). Physical examination revealed proportionate short stature with no bone deformity. Her pubic hair and breasts were both at Tanner stage I, and her sex hormones were normal with respect to gender and age. Ultrasound imaging of the heart, carotid and vertebral arteries showed no abnormalities. In the insulin-induced hypoglycemia provocative test, her GH peaked at 4.99 ng/mL. A low GH peak (2.80 ng/mL) was also observed in the L-dopa GH provocative test (**Supplementary Figure 1**). In addition, this patient had low-normal serum IGF-1 (138 ng/mL, SDS = -1.16, age-specific normal range: 74-388 ng/mL). Her IGFBP3 (4.1 ug/mL, age-specific normal range: 3.9-11.7 ug/ml, SDS = -1.86) was below -1.80 SDS, the best cutoff point for differentiating childhood-onset GHD from ISS as described by Buoquete et al. (18). Moreover, her growth chart exhibited progressive growth failure before rhGH administration, which was typically observed in GHD patients. Furthermore, her height increased 9.6 cm within 8 months after receiving rhGH at a dose of 2.0 U qn [from 124.2 cm (SDS = -2.94) to 133.8 cm (SDS = -1.94)]. Her clinical features, including a peak GH below 7 ng/mL in two GH stimulation tests, IGFBP3 SDS < -1.80, growth faltering at diagnosis and good response to rhGH therapy, all strongly supported the diagnosis of GHD.

**Figure 2:**
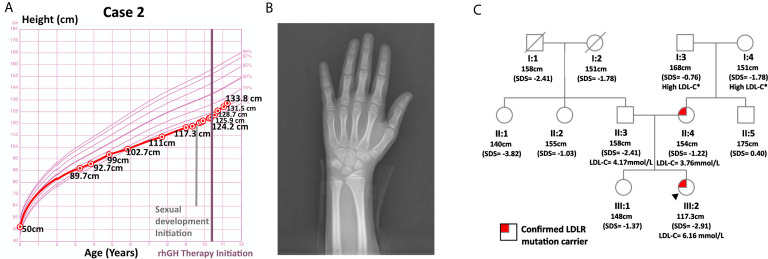
Information on case 2. **(A)** Growth curve. **(B)** Plain X-ray of left hand and wrist. **(C)** Pedigree.

Her TC (7.70 mmol/L) and LDL-C (6.16 mmol/L) were significantly elevated, while HDL-C (1.24 mmol/L), TG (0.80 mmol/L), fasting blood glucose (4.7 mmol/L), 25(OH)D and thyroid hormones were normal. She had a positive family history of hypercholesterolemia as both her parents and grandparents on her mother’s side suffered from high LDL-C. WES did not detect any GHD-related mutations in case 2. However, it spotted a heterozygous variant of the *LDLR* gene, c.809 G > A (p.Cys270Tyr, Ensembl transcript ID of the reference sequence: NM_ 000527.4), which was likely pathogenic (21, 22). Sanger sequencing confirmed the presence of this mutation and revealed that the patient inherited this mutation from her mother. While both this patient and her mother were heterozygous for this mutation, her father and healthy sister were normal at this site (**Figure 2**). This mutation was deposited in disease databases like ClinVar and the Human Gene Mutation Database (HGMD) as a likely pathogenic variant. It was also considered to be disease-causing by bioinformatic tools like MutationTaster and PolyPhen-2 due to its potential harmful effects on the protein structure (23, 24). As shown in **Supplementary Figure 2**, this mutation may hamper the ligand binding capacity of the LDL receptor.

### Literature Review Results

**Figure 3:**
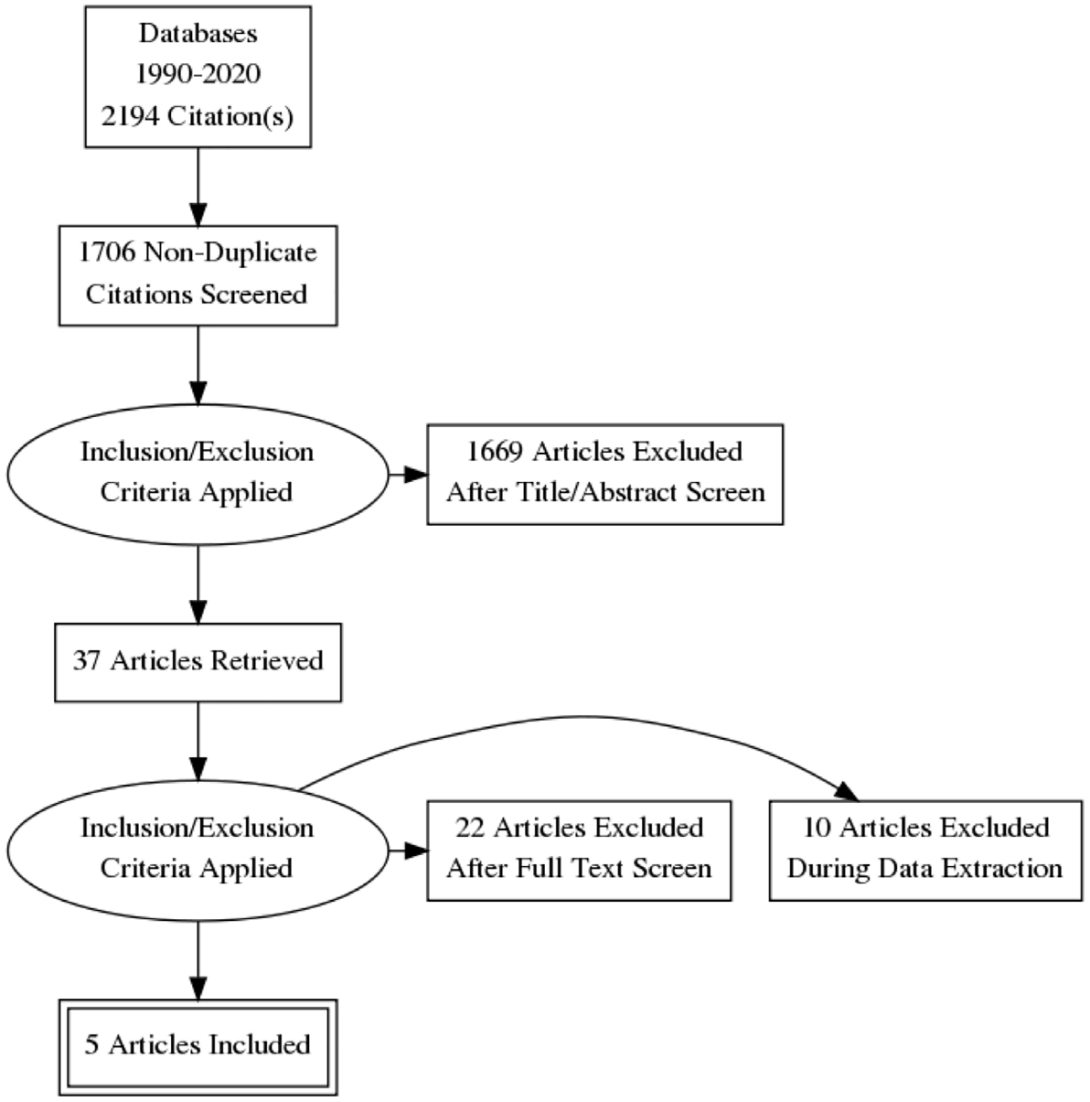
Flow diagram of study selection.

**Figure 4:**
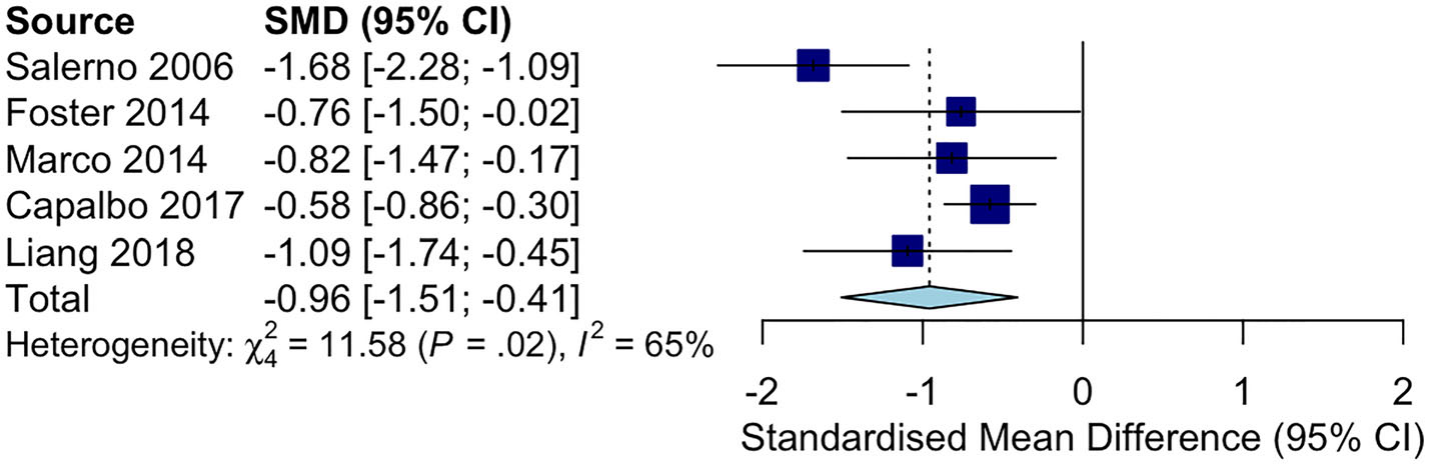
Forest plot comparing ΔLDL-C in GHD patients treated with rhGH v.s. controls not receiving any treatments. (ΔLDL-C = LDL-C at the end point - baseline LDL-C).

The following Preferred Reporting Items for Systematic Reviews and Meta-Analyses (PRISMA) diagram illustrates the study selection procedure (**Figure 3**). From 1706 potentially relevant studies, 1669 were excluded after screening the title and the abstract. 37 studies were retrieved for detailed assessment: 32 were excluded due to the lack of control group or control group lipid data at the end point. As a result, we found 5 trials that were eligible for inclusion (25–29), which involved 191 patients in the treatment group and 183 people in the control group. All of these trials were observational studies, and we did not identify any randomized controlled trials on this topic. Spanning from 6 months to 2 years, rhGH therapy effectively reduced the LDL-C level (SMD = -0.96, 95% CI: -1.51 to -0.41, P = 0.02, I^2^ = 65%) (**Figure 4**) in GHD patients.

## Discussion

Both of these 2 cases were diagnosed of GHD according to short stature and low peak GH level in GH provocative test. It is reasonable to state that the insufficiency of GH in these two patients exert negative effects on their blood cholesterol. Partially deprived of GH and IGF-1, GHD patients generally have higher LDL-C level than age, race and sex matched healthy children. While the mean LDL-C of GHD children typically ranged from 2.39 to 3.89 mmol/L, the mean LDL-C of non-GHD controls was between 1.73 and 2.58 mmol/L (**Supplementary Table 2**).

However, the LDL-C of these two cases was apparently higher than the average LDL-C of GHD children. Hence, low GH level was unlikely to be the only reason that could fully account for their elevated blood cholesterol. In case 1, laboratory tests revealed TC of 7.08 mmol/L and LDL-C of 4.80 mmol/L. In addition, the patient inherited a pathogenic copy of the *APOB* gene from his mother. *APOB* gene guides the synthesis of apolipoprotein B-48 and apolipoprotein B100 (apoB100). The replacement of arginine with tryptophan reduces apoB100’s binding affinity with the LDL receptor, resulting in prolonged circulation time of LDL particles (30). In terms of the second case, her TC was 7.70 mmol/L and her LDL-C was 6.16 mmol/L. Moreover, she had a mutated allele of the *LDLR* gene. In specific, the *LDLR* gene encodes the LDL receptor that mediates the endocytosis of LDL particles, thereby reducing the LDL-C burden in the blood stream. In summary, elevated LDL-C level together with genetic mutation detected in the LDL-C metabolic pathway confirmed the diagnosis of definite FH in both cases according to the Dutch Lipid Clinic Network (DLCN) criteria (6).

The hypercholesterolemic state in these two cases may negatively influence their growth and development. In FH patients, cells have limited capability to take up cholesterol from the blood stream. The inefficacy of utilizing blood cholesterol may impair cell growth and anabolism. Moreover, persisting hypercholesterolemia may lead to cholesterol accumulation in the anterior pituitary gland and alter the morphology of pituitary cells. For instance, rats on high cholesterol diet exhibited a decreased proportion of acidophilic cells and an increased percentage of basophilic cells as compared with counterparts on normal diet (31). As somatotrophs belong to the group of acidophilic cells, the secretion of GH may be compromised. Furthermore, hyperlipidemia may promote endoplasmic reticulum stress and activate the IKKβ/NF-κB pathway in the hypothalamus (32). Chronic metabolic inflammation may interfere with the endocrine function of the hypothalamus, including major endocrine axes such as the hypothalamus-somatotroph axis (33). Given the belief that elevated blood cholesterol may affect the growth of children, guidelines for the management of pediatric FH actively recommend for monitoring the height, weight and puberty development of FH children (34). In essence, the relation between GHD and FH is likely to be bidirectional. While the deficiency of GH aggravates hypercholesterolemia, the secretion and function of GH and IGF-1 might be negatively affected by high blood cholesterol level. Nevertheless, hitherto the effects of FH on GH level and height velocity have not been quantitively examined. It is worthwhile to compare the height, peak GH, and IGF-1 concentration of FH children with age, sex and race matched subjects.

Therefore, these two cases indicate that GHD patients with high LDL-C should be assessed for FH. As cumulative LDL-C burden results in severe cardiovascular complications, the early detection and treatment of FH are important for delaying the progression of atherosclerosis and ensuing cardiovascular events. However, since phenotypes are mild in the first two decades of life, FH remains largely underdiagnosed and undertreated before the onset of the first cardiovascular event (6). In practice, the lipidomic profiles of FH patients could significantly deteriorate if GHD coexists. For example, Merimee studied 6 hypercholesterolemia families and revealed that family members with GHD had significantly elevated LDL-C as compared with non-GHD relatives (35). Hence, screening GHD patients with hypercholesterolemia for lipid metabolic disorders may aid the early diagnosis of FH.

As shown in **Figure 4**, rhGH therapy effectively reduced the LDL-C level (SMD = -0.96, 95% CI: -1.51 to -0.41, P = 0.02, I^2^ = 65%) in GHD patients. Moreover, Lind et al. observed that rhGH was also effective in reducing LDL-C in HeFH patients. In particular, the average LDL-C of 8 middle-aged men with HeFH (BMI: 25.0 ± 0.95, slightly overweight on average) decreased from 8.7 to 7.4 mmol/L after receiving rhGH at the dose of 0.1 IU/kg/d for 3 weeks (8). Growth hormone stimulates the clearance of LDL-C by upregulating liver *LDLR* mRNA expression, accelerating LDL-C catabolism and reducing the lipid content of tissues (36). Hence, the extent of improvement in blood cholesterol following rhGH therapy largely depends on the residual activity of *LDLR*. HeFH patients’ lipid profile can be significantly improved by rhGH owing to the residual activity of their mutated LDL receptors. In homozygous FH (HoFH), the LDL receptor is so defective that it largely remains inactive even when stimulated by rhGH (8). Additionally, the effectiveness of rhGH therapy in reducing LDL-C in FH patients is affected by genetic polymorphism in lipid metabolism. According to Barbosa et.al, APOB SNP rs676210 GG genotype is associated with more significant reduction in LDL-C during rhGH therapy (37). GH receptor variants also considerably affect serum lipid levels in GHD patients. Ihara et al. illustrated that GHD boys who harbored the p.Leu544IIe variant in GH receptor had higher TC at baseline (38). These patients successfully gained height after receiving rhGH therapy, but their TC remained high. Furthermore, the serum non-HDL cholesterol levels under GH deficient conditions were modulated by variants in genes that regulate lipogenic pathways, such as the *signal transducer and activator of transcription 5A/B (STAT5A/B)*, and the *activating transcription factor 6 (ATF6)* gene (39, 40). Generally, the use of rhGH in HeFH children may help keep their LDL-C level in control while reducing the dose of standard LDL-C lowering agents such as statins and ezetimibe.

In terms of therapeutic interventions, the Integrated Guidelines for Cardiovascular Health and Risk Reduction in Children and Adolescents recommend children to initiate cholesterol-lowering therapy after a 6-month trial of lifestyle changes if they fall into one of the following categories: 1) ≥ 10 years old and LDL-C ≥ 190 mg/dL (4.9 mmol/L); 2) ≥ 10 years old, LDL-C below 190 mg/dL but higher than 160 mg/dL (4.1 mmol/L), and positive family history of premature CVD; 3) ≥ 8 years old, LDL-C ≥ 190 mg/dL, and positive family history of early-onset CVD; or 4) homozygous FH (41). However, there is not a clear-cut for the age to start lipid-lowering medication. In particular, bile acid sequestrants were proved by cohort studies to be safe for children as young as 6 years old, while statins were safely used in children ≥ 8 years old (7). LDL-C is preferably kept ≤ 130 mg/dL (3.36 mmol/L) in pediatric patients, while < 110 mg/dL (2.85 mmol/L) is even more ideal when other CVD risk factors coexist (41). In terms of our first patient, his lipid profile improved after going on a low fat diet and doing regular exercise for 6 months. Specifically, his TC decreased to 6.9 mmol/L, and his LDL-C dropped to 4.5 mmol/L (**Supplementary Figure 2**). Given his young age and the effectiveness of lifestyle management, we encouraged him to continue with lifestyle control and height velocity monitoring, rather than hastily starting cholesterol-lowering medication. As for the second case, lipid-lowering therapy was initiated at the age of 10 after she failed a 9-month trial of lifestyle adjustment (TC = 7.73 mmol/L, LDL-C = 6.05 mmol/L after lifestyle management). Ezetimibe was administered at first, but her lipid levels were still high at a daily dose of 10 mg ezetimibe (TC: 5.66 mmol/L, LDL-C: 4.09 mmol/L). Hence, we shifted her lipid-lowering agent to atorvastatin. After taking atorvastatin (10 mg qd) for one month, her LDL-C was roughly controlled (3.84 mmol/L). Then, rhGH therapy was started and a growth spurt was observed. As previously mentioned, her height increased 9.6 cm after receiving rhGH at a dose of 2.0U qn for 8 months [from 124.2 cm (SDS = -2.94) to 133.8 cm (SDS = -1.94)]. As oxidized LDL-C was proved to promote atherosclerotic plaque formation and an abrupt increase in GH may accelerate LDL-C oxidation (42), we decided to keep the patient’s LDL-C under control before initiating rhGH therapy. According to **Supplementary Figure 1**, after taking atorvastatin for about 10 months and rhGH for approximately 6 months, the patient’s serum cholesterol was adequately controlled (TC: 4.90 mmol/L, LDL-C: 3.22 mmol/L). We advocate for setting out evidence-based clinical guidelines for treating children with coexisting GHD and FH. It is also helpful to study whether lipid-controlling agents should precede rhGH therapy or these two treatments can be concurrently initiated.

Potential limitations of this work warrant consideration. WES may not detect large copy number variants (CNVs). Therefore, multiple ligation-dependent probe amplification (MLPA) or chromosomal microarray (CMA) analysis should be performed to screen for large deletions and insertions in genes belonging to the short stature gene panel. Moreover, the effects of LDL-C on GH level have not been validated by experiments. Additional research is needed to fully understand the mechanisms underlying the correlation between LDL-C and GH. Furthermore, for the second patient, screening relatives on her mother’s side for the c.809 G >A mutation can provide more evidence on the pathogenicity of this *LDLR* variant. Co-segregation of this mutation and hypercholesterolemia in a large pedigree can strongly support its pathogenicity. Lastly, the average LDL-C level in patients with coexisting GHD and FH deserves further study in a larger cohort.

## Conclusion

We present 2 intriguing cases of pediatric GHD with FH. Routinely screening for dyslipidemia in GHD patients may facilitate the early detection of FH. FH should be suspected when the remarkably high LDL-C level of GHD patients cannot be explained by GHD alone.

## Patient’s Perspective

First case: not available.

Second case: “After having the daily injection of rhGH, my appetite slightly increased. I am so surprised that I can gain height at a speed that I had never imaged. However, the injection of rhGH is a little bit painful and troublesome. I would be happier if rhGH could be taken orally. The doctor suggested me to eat healthier food and exercise daily, which could be hard as I have no other choices but to have lunch at school in the weekdays”.

Her mother: “I was worried about the side effects of lipid-lowering therapy. However, I was convinced that the benefits of taking atorvastatin outweighed the disadvantages. Luckily, my daughter said that she felt well and the lab test results were roughly normal”.

## Data Availability Statement

The original contributions presented in the study are included in the article/**Supplementary Material**. Further inquiries can be directed to the corresponding author.

## Ethics Statement

The studies involving human participants were reviewed and approved by Institutional Review Committee of Peking Union Medical College Hospital. Written informed consent to participate in this study was provided by the participants’ legal guardian/next of kin.

## Author Contributions

SY and HZ conceived the idea and conceptualized the study. SY drafted the manuscript. HZ reviewed the manuscript. SY and XK performed literature review and analyzed the data. HL and RL performed genetic analysis of the patients. All authors contributed to the article and approved the submitted version.

## Funding

This work was supported by the Chinese Academy of Medical Sciences-CAMS Innovation Fund for Medical Sciences (CAMS-2016-I2M-1-002) and the National Key Research and Development Program of China (No. 2016YFC091500).

## Conflict of Interest

The authors declare that the research was conducted in the absence of any commercial or financial relationships that could be construed as a potential conflict of interest.
