# Supplementary material for: Case Report: A Clinical and Genetic Analysis of Childhood Growth Hormone Deficiency With Familial Hypercholesterolemia
Source: Front Endocrinol (Lausanne). 2021 Jun 18;12:691490. doi: 10.3389/fendo.2021.691490 (PMC8249922; doi:10.3389/fendo.2021.691490)
Supplement: Supplementary file 1 [file Table_1.docx]

Supplementary Material

# Supplementary Tables

## Supplementary Table 1. Detailed search strategy

| # | Term |
| --- | --- |
| #1 | “Child”[Mesh] |
| #2 | children |
| #3 | childhood |
| #4 | pediatri* |
| #5 | kid |
| #6 | infant |
| #7 | “Adolescent”[Mesh] |
| #8 | teenager |
| #9 | juvenile |
| #10 | #1 OR #2 OR #3 OR #4 OR #5 OR #6 OR #7 OR #8 OR #9 |
| #11 | GHD |
| #12 | growth hormone deficiency |
| #13 | short stature |
| #14 | #11 OR #12 OR #13 |
| #15 | #10 AND #14 |
| #16 | “Human Growth Hormone”[Mesh] |
| #17 | rhGH |
| #18 | recombinant human growth hormone |
| #19 | somatotropin |
| #20 | #16 OR #17 OR #18 OR #19 |
| #21 | treat* |
| #22 | therapy |
| #23 | medication |
| #24 | supplement |
| #25 | replacement |
| #26 | #21 OR #22 OR #23 OR #24 OR #25 |
| #27 | #20 AND #26 |
| #28 | “Lipids”[Mesh] |
| #29 | “Fats”[Mesh] |
| #30 | “Hyperlipidemia”[Mesh] |
| #31 | “Dyslipidemia”[Mesh] |
| #32 | metabolism |
| #33 | cholesterol |
| #34 | lipoprotein |
| #35 | LDL-C |
| #36 | #28 OR #29 OR #30 OR #31 OR #32 OR #33 OR #34 OR #35 |
| #37 | RCT |
| #38 | randomized controlled trial |
| #39 | controlled clinical trial |
| #40 | cohort |
| #41 | case control |
| #42 | groups |
| #43 | #37 OR #38 OR #39 OR #40 OR #41 OR #42 |
| #44 | #15 AND #27 AND #36 AND #43 |

## Supplementary Table 2. Characteristics of studies included in the analysis of LDL-C in GHD patients

| Author | Year | Sample size | | Age (years) | | rhGH dose | Duration  (months) | Control type | Study type |
| --- | --- | --- | --- | --- | --- | --- | --- | --- | --- |
|  |  | Treatment group | Control group | Treatment group | Control group |  |  |  |  |
| Salerno (1) | 2006 | 30 | 30 | 9.3±0.5 | 9.8±0.6 | 30ug/kg/day | 24 | healthy normal | Case-control |
| Foster (2) | 2014 | 18 | 13 | 10.4±2.0 | 10.3±1.8 | 0.25-0.3mg/kg/week | 6 | short normal | Case-control |
| Marco (3) | 2014 | 20 | 20 | 9.5±1.8 | 8.8±1.5 | 0.035mg/kg/day | 12 | healthy normal | Case-control |
| Capalbo (4) | 2017 | 100 | 100 | 9.42±3.65 | 8.96±2.82 | 25-30mg/kg/day | 12 | healthy normal | Case-control |
| Liang (5) | 2018 | 23 | 20 | 11.61±1.73 | 11.25±1.86 | 0.23-0.35mg/kg/week | 6 | GHD patient | Case-control |

| Baseline LDL-C (mmol/L) | | LDL-C at the end (mmol/L) | | ΔLDL-C* (mmol/L) | |
| --- | --- | --- | --- | --- | --- |
| Treatment group | Control group | Treatment group | Control group | Treatment group | Control group |
| 2.5±0.2 | 2.1±0.3 | 2.1±0.2 | 2.1±0.1 | -0.4±0.2 | 0±0.26 |
| 2.39±0.69 | 2.46±0.53 | 2.39±0.58 | 2.95±0.66 | 0±0.64 | 0.49±0.61 |
| 2.88±0.57 | 2.2±0.41 | 2.47±0.83 | 2.22±0.55 | -0.41±0.53 | 0.02±0.5 |
| 2.53±0.66 | 2.29±0.72 | 2.2±0.63 | 2.33±0.4 | -0.33±0.65 | 0.04±0.62 |
| 2.87±0.47 | 2.58±0.85 | 2.2±0.45 | 2.63±0.76 | -0.67±0.46 | 0.05±0.81 |

*The standard deviation of the change in LDL-C from baseline to the end of the study was estimated by the following equation if it was not provided: ${SD}_{change}=\sqrt{{({SD}_{baseline})}^{2}+{({SD}_{endpoint})}^{2}-2\times r\times{SD}_{baseline}\times{SD}_{endpoint}}$ , where r represents the correlation coefficient. We took r = 0.5 as a conservative estimate in the analysis.

## Supplementary Table 3. Detailed Newcastle-Ottawa Scale for each included study

|  | Selection | | | | Comparability | | Outcome | | | |
| --- | --- | --- | --- | --- | --- | --- | --- | --- | --- | --- |
| Study | Case definition adequate | Representativeness of the cases | Selection of controls | Definition of controls | Controls for the most important factor | Adjust for additional factors | Assessment of exposure | Same method of ascertainment for cases and controls | Non-Response rate | Total quality score |
| Salerno | 1 | 1 | 0 | 0 | 0 | 1 | 1 | 1 | 1 | 6 |
| Foster | 1 | 1 | 1 | 1 | 1 | 0 | 1 | 1 | 1 | 8 |
| Marco | 1 | 1 | 0 | 0 | 0 | 1 | 1 | 1 | 1 | 6 |
| Capalbo | 1 | 1 | 0 | 0 | 0 | 1 | 1 | 1 | 1 | 6 |
| Liang | 1 | 1 | 1 | 1 | 1 | 1 | 1 | 1 | 1 | 9 |

# Supplementary Figures


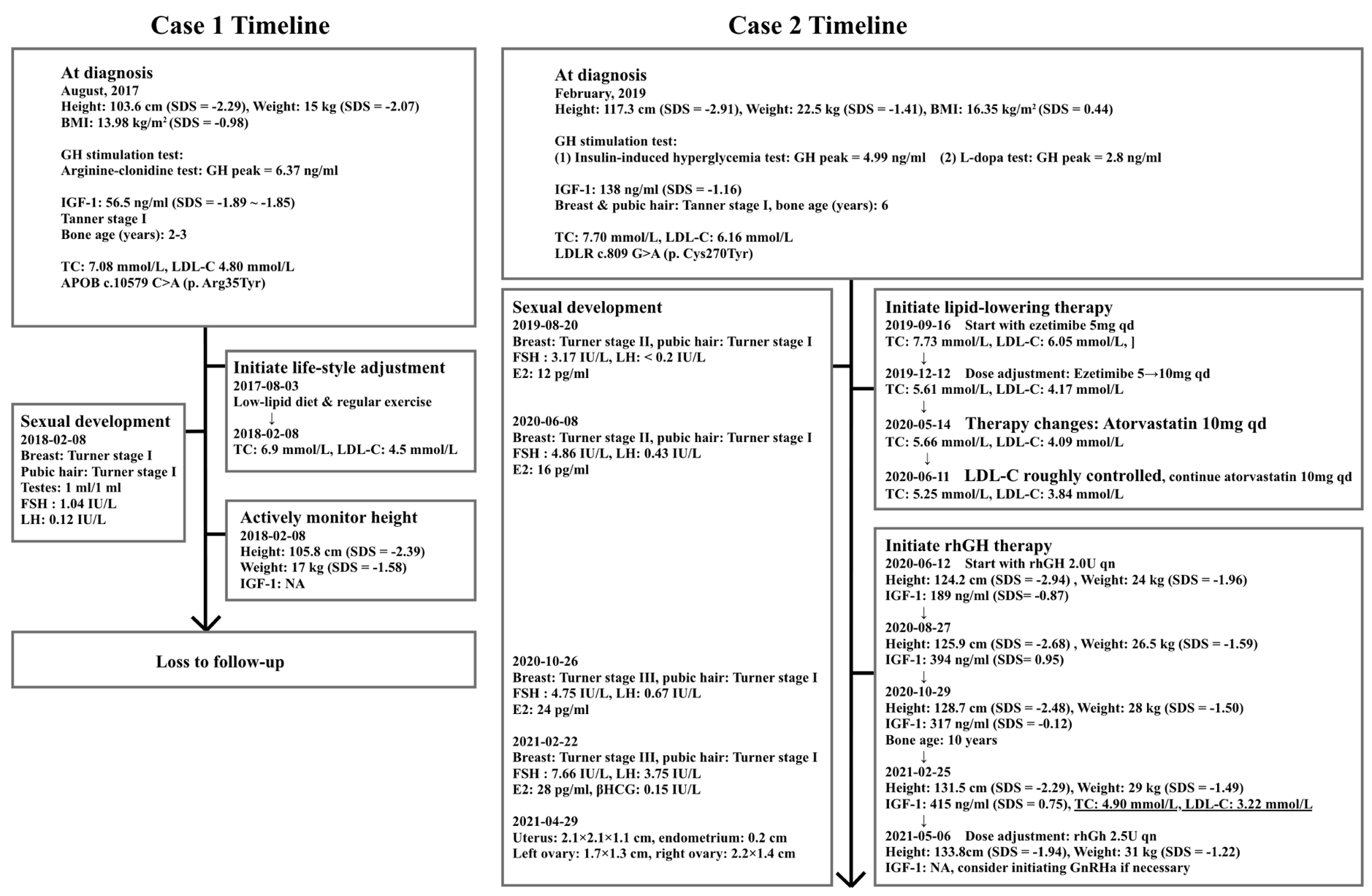


**Supplementary Figure 1.** Timeline for episode of care.


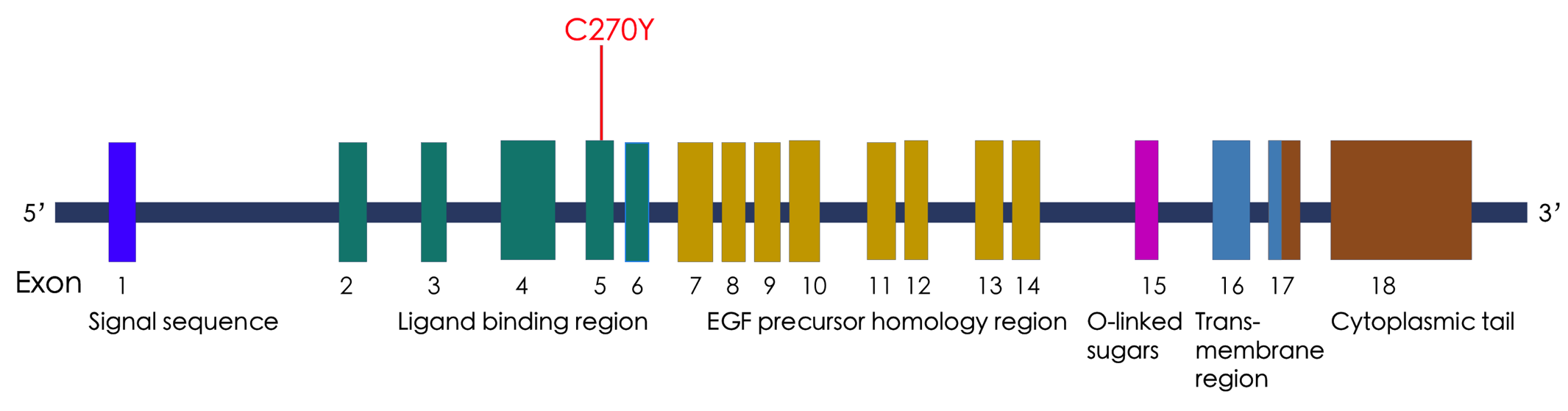


**Supplementary Figure 2.** Diagram of the *LDLR* gene showing the mutation detected in case 2. Exons are represented by vertical boxes and introns are denoted as regions connecting the exons.

# References

1. Salerno M, Esposito V, Farina V, Radetti G, Umbaldo A, Capalbo D, et al. Improvement of cardiac performance and cardiovascular risk factors in children with GH deficiency after two years of GH replacement therapy: an observational, open, prospective, case-control study. J Clin Endocrinol Metab. 2006;91(4):1288-95. doi: 10.1210/jc.2005-0981

2. Foster C, Burton A, Scholl J, Scott ML, Gunter V, McCormick K. Lipid patterns in treated growth hormone deficient children vs. short stature controls. J Pediatr Endocrinol Metab. 2014;27(9-10):909-14. doi: 10.1515/jpem-2013-0488

3. De Marco S, Marcovecchio ML, Caniglia D, De Leonibus C, Chiarelli F, Mohn A. Circulating asymmetric dimethylarginine and lipid profile in pre-pubertal children with growth hormone deficiency: effect of 12-month growth hormone replacement therapy. Growth Horm IGF Res. 2014;24(5):216-20. doi: 10.1016/j.ghir.2014.08.001

4. Capalbo D, Esposito A, Improda N, Wasniewska MG, Di Mase R, De Luca F, et al. Glucose homeostasis in GHD children during long-term replacement therapy: a case-control study. Endocrine. 2018;59(3):643-50. doi: 10.1007/s12020-017-1408-0

5. Liang S, Xue J, Li G. Effects of recombinant human growth hormone administration on cardiovascular risk factors in obese children with relative growth hormone deficiency. Lipids Health Dis. 2018;17(1):66. doi: 10.1186/s12944-018-0721-9
